# Lifestyle-Based Approaches to Cancer Prevention and Treatment: Diet, Physical Activity, and Integrative Strategies

**DOI:** 10.3390/pathophysiology32040070

**Published:** 2025-12-17

**Authors:** Gianpiero Greco, Alessandro Petrelli, Francesco Fischetti, Stefania Cataldi

**Affiliations:** 1 Department of Translational Biomedicine and Neuroscience (DiBraiN), University of Study of Bari, 70124 Bari, Italy; alessandro.petrelli@uniba.it (A.P.); francesco.fischetti@uniba.it (F.F.); 2 Department of Neurosciences, Biomedicine and Movement Sciences, University of Verona, 37129 Verona, Italy; 3 Department of Education and Sport Sciences, Pegaso Telematic University, 80143 Naples, Italy; stefania.cataldi@unipegaso.it

**Keywords:** carcinogenic exposures, fasting-mimicking diets, ketogenic strategies, metabolic health, exercise oncology, ultraviolet radiation, complementary therapies, survivorship

## Abstract

Cancer remains a leading global cause of morbidity and mortality. Modifiable lifestyle factors, including avoidance of tobacco use and excessive ultraviolet radiation, healthy dietary patterns, regular physical activity, and weight management, play key roles in prevention and care. This narrative review synthesizes evidence on lifestyle-based interventions influencing cancer risk, treatment tolerance, and survivorship. A literature search was conducted in PubMed and Scopus, supplemented by manual screening via Google Scholar. The time frame (2001–2025) was selected to reflect evidence produced within the modern era of molecular oncology and contemporary lifestyle medicine research. Eligible publications addressed carcinogen exposure (tobacco, alcohol, ultraviolet radiation), diet and nutritional strategies, physical activity, sedentary behavior, obesity, metabolic health, complementary therapies, and cancer outcomes. Evidence indicates that reducing exposure to tobacco and ultraviolet radiation remains central to cancer prevention. Adherence to predominantly plant-based diets, regular physical activity, and maintenance of healthy body weight are consistently associated with lower incidence of several cancers, including breast, colorectal, and liver cancer. Nutritional strategies such as caloric restriction, ketogenic diets, and fasting-mimicking diets show promise in improving treatment efficacy and quality of life. Complementary and mind–body therapies may alleviate treatment-related symptoms, although high-quality evidence on long-term safety and effectiveness is limited. Integrating lifestyle medicine into oncology offers a cost-effective, sustainable strategy to reduce cancer burden and enhance survivorship. Comprehensive programs combining carcinogen avoidance, dietary regulation, structured exercise, and effective radiation risk mitigation may extend healthspan, improve treatment tolerance, and help prevent recurrence.

## 1. Introduction

Despite notable advances in cancer detection and treatment, completely eradicating the burden of cancer remains an unlikely outcome. Current cancer control strategies rely on early detection, surgery, pharmacological therapy, radiotherapy, and targeted or immunotherapeutic approaches. While these interventions remain essential pillars of modern oncology, their effectiveness is often limited by late diagnosis, variability in treatment response, and therapy-related toxicity [1,2,3]. Against this backdrop, lifestyle medicine—integrating diet, physical activity, and complementary mind–body strategies—has emerged as a cost-effective and accessible adjunct to conventional cancer care, with potential benefits in both prevention and survivorship [1,4,5].

A growing body of epidemiological and mechanistic evidence supports these lifestyle-based pillars. Dietary patterns rich in plant-based foods and low in ultra-processed products have been consistently associated with reduced incidence of breast, colorectal, liver, and other cancers, partly through modulation of inflammation, metabolic signaling, and insulin-like growth factor pathways [1,2,3,4,5,6]. Regular physical activity independently lowers the risk of several major cancers and exerts beneficial effects on insulin sensitivity, immune regulation, systemic inflammation, and metabolic homeostasis [6,7]. Integrative and mind–body approaches—such as meditation, yoga, Tai Chi, and selected complementary therapies—are increasingly adopted to alleviate treatment-related side effects, with evidence suggesting improvements in fatigue, anxiety, and overall quality of life [8,9,10,11,12,13,14]. These data collectively justify the focused examination of diet, physical activity, and integrative strategies as complementary components of contemporary cancer prevention and care.

A rational strategy for cancer control involves prioritizing primary prevention while also advancing new therapies and improving early detection. Promoting healthy lifestyle choices is central to effective primary prevention and lowering cancer risk. This includes adopting a predominantly plant-based diet, sustaining healthy body weight over time, engaging in consistent physical activity, avoiding or minimizing tobacco and alcohol use, and reducing exposure to radiation and carcinogenic substances. These preventive behaviors are expected to significantly lower the incidence of several cancers, such as melanoma, breast, colorectal, esophageal, liver, and head and neck cancers [1].

Specific lifestyle recommendations for preventing melanoma include consistent sunscreen use, wearing protective clothing, seeking shade, and limiting sun exposure during periods of peak ultraviolet radiation, which generally occur between late morning and mid-afternoon depending on geographical latitude and season (often within the 10 a.m.–4 p.m. window in many regions), as well as avoiding indoor tanning [2]. The role of carotenoid intake, particularly β-carotene and vitamin A, in cancer prevention has been explored, although findings remain mixed. While observational studies report inverse associations between dietary carotenoid intake and several cancers, large trials have shown that high-dose β-carotene supplementation may increase lung cancer risk among smokers, underscoring the importance of distinguishing food-based intake from pharmacological supplementation [3]. This distinction is particularly relevant given the divergent biological effects of carotenoids when consumed in whole foods versus isolated high-dose supplements. Around 90% of lung cancer cases are attributed to smoking, and the combined use of tobacco and alcohol further elevates the risk of developing cancer. Globally, the use of smokeless tobacco is linked to approximately 400,000 cases of oral cancer, accounting for 4% of all cancers [4]. Therefore, tobacco cessation and alcohol moderation are likely to play a critical role in reducing cancer incidence.

There is a well-established connection between dietary habits, obesity, metabolic syndrome, and the development of various cancers. These factors are estimated to contribute to about 30–35% of cancer-related deaths, suggesting that many of these could be preventable through dietary interventions. Such approaches include fasting, fasting-mimicking diets, ketogenic diets, and calorie restriction [5]. Recent studies have also examined the impact of dietary advanced glycation end products (AGEs) on cancer risk. Although the overall evidence remains inconclusive, higher AGE intake has been linked to increased oxidative stress and inflammation, potentially elevating the risk of certain malignancies, such as breast cancer [15,16,17].

Emerging research also highlights the synergistic potential of combining energy restriction—whether dietary or pharmacological—with regular physical activity to promote healthy aging and reduce cancer risk. These combined strategies have been shown to modulate key biological pathways involved in carcinogenesis, including inflammation and metabolic dysregulation [6].

This narrative review focused on lifestyle-based interventions aimed at improving cancer prevention and reducing associated risks, while also enhancing the overall health of cancer patients. Additionally, it explores the therapeutic potential of specific dietary strategies, nutritional supplements, and complementary and alternative medicine in cancer care.

## 2. Search Strategy and Literature Selection

This narrative review was developed by examining peer-reviewed literature relevant to lifestyle-based approaches in cancer prevention, treatment tolerance, and survivorship. A broad, non-systematic search of scientific literature was performed using PubMed and Scopus, complemented by manual searches in Google Scholar to identify additional relevant articles. The search was completed on 11 August 2025 and focused on publications from 2001 to 2025. This 25-year window was chosen to capture contemporary evidence generated in the era of modern molecular oncology, exercise oncology, and integrative medicine; while excluding older studies whose diagnostic criteria, treatment protocols, and exposure assessments are less comparable with current clinical practice.

The search combined terms related to cancer and oncology with terms describing lifestyle factors (dietary patterns, nutritional interventions, physical activity, exercise, body weight, obesity), complementary and integrative approaches (mind–body therapies, complementary and alternative medicine, integrative oncology), carcinogenic exposures (tobacco, alcohol, radiation), specific nutritional strategies (fasting protocols, caloric restriction, ketogenic diets, vitamins, carotenoids), and major cancer types (e.g., breast, colorectal, liver, prostate, lung, endometrial cancer, and melanoma).

Studies were considered for inclusion if they addressed at least one lifestyle-related factor in the context of cancer prevention, treatment response, or survivorship and were published in English in peer-reviewed journals. Experimental animal studies, articles focusing exclusively on pediatric or veterinary populations, preprints and other non–peer-reviewed reports, and papers clearly outside the scope of lifestyle-based cancer prevention or management were not retained. In keeping with the narrative design, no formal risk-of-bias assessment was conducted; however, study quality was indirectly taken into account by prioritizing higher-level evidence (systematic reviews, randomized controlled trials, and large cohort studies), without applying journal-level metrics such as Journal Citation Reports quartiles. A concise overview of the search approach is provided in the Supplementary Materials, Table S1.

## 3. Results and Discussion

This section synthesizes the evidence identified in the literature search and presents it in a narrative format, consistent with the aims of a non-systematic review. Findings are organized to first describe major carcinogenic exposures relevant to cancer risk (tobacco use and ultraviolet radiation), followed by the three core lifestyle pillars addressed in this review: (i) dietary strategies, (ii) physical activity, sedentary behavior, and exercise oncology, and (iii) complementary and mind–body approaches. Evidence from observational studies, randomized and non-randomized clinical trials, and mechanistic research is integrated to provide a comprehensive interpretation of how these factors influence cancer prevention, treatment tolerance, and survivorship.

### 3.1. Carcinogenic Exposures: Tobacco and Ultraviolet (UV) Radiation

#### 3.1.1. Tobacco as a Carcinogen

Tobacco and tobacco smoke contain a complex mixture of over 9500 chemical substances, many of which have been identified by regulatory authorities as harmful to human health. In a 2022 update, Li and Hecht reviewed the major carcinogenic constituents of tobacco and tobacco smoke, including nitrosamines, polycyclic aromatic hydrocarbons, and aromatic amines, emphasizing their role in cancer development [18]. Tobacco smoke increases cancer risk primarily due to its content of carcinogens such as nitrosamines, acrylamides, polycyclic aromatic hydrocarbons (PAHs), cadmium, and volatile organic compounds (VOCs) [19,20].

There is a well-established causal link between smoking and lung cancer risk [19]. Extensive experimental and epidemiological evidence confirms that smoking is a major risk factor for several cancers, including those of the kidney, bladder, gastrointestinal tract, head and neck, colorectum, esophagus, pancreas, stomach, liver, cervix, and myeloid leukemia [18,19,21,22,23].

Animal studies have shown that several carcinogens in tobacco smoke can induce mammary tumors, and human research has demonstrated that various tobacco-derived compounds can reach breast tissue. Furthermore, the absorption and metabolic activation of carcinogens implicated in breast cancer—such as 4-aminobiphenyl and PAHs—are significantly higher in smokers than in non-smokers [22].

Head and neck squamous cell carcinoma (HNSCC) is a cancer type strongly associated with tobacco use. The risk of developing HNSCC is nearly ten times higher in smokers compared to non-smokers, and approximately 70–80% of newly diagnosed cases are linked to the combined use of tobacco and alcohol [23].

The carcinogenic effects of tobacco smoke are largely mediated by reactive oxygen species (ROS) and reactive nitrogen species (RNS), which damage key macromolecules such as DNA, lipids, and proteins. Scientific evidence indicates that smoking-induced oxidative stress plays a central role in both cancer development and inflammation. This oxidative stress triggers inflammatory responses, which in turn generate additional ROS, perpetuating a cycle of molecular damage that may initiate carcinogenesis [24].

#### 3.1.2. Non-Ionizing Radiation as a Carcinogen

Human exposure to radiofrequency electromagnetic fields (RF-EMF) primarily arises from the use of personal electronic devices—such as mobile phones, Bluetooth headsets, cordless phones, and amateur radios—as well as from occupational and environmental sources. Occupational exposure includes equipment like induction heaters, high-frequency dielectric devices, and high-powered pulsed radars, while environmental sources involve medical technologies, broadcast antennas, and mobile phone base stations. Among the general population, the highest levels of exposure typically come from handheld devices used in close proximity to the body, particularly mobile phones. Additionally, numerous occupational groups are regularly exposed to RF radiation, including radar operators, military and security personnel using walkie-talkies, technicians servicing broadcast antennas, plastic welders, workers involved in RF-based testing or drying processes, and physiotherapists using diathermy equipment [25].

Stang et al. conducted both hospital- and population-based case–control studies investigating the development of uveal melanoma in relation to occupational RF radiation exposure and reported an increased risk among individuals exposed to radiofrequency-emitting devices, such as radios and mobile phones [26]. Similarly, Karipidis et al. carried out a case–control study across five major population centers in Victoria, Australia, identifying a potential link between occupational RF exposure and glioma risk [27].

Ultraviolet (UV) radiation, a non-visible component of the electromagnetic spectrum with shorter wavelengths than visible light, is also recognized as carcinogenic. While sunlight is the primary source of UV radiation, certain industrial activities—such as electric arc welding—can also emit significant UV levels. The International Commission on Non-Ionizing Radiation Protection (ICNIRP) has established a safety threshold for artificial UV exposure to the eyes and unprotected skin, recommending a limit of 30 J/m^2^ over an 8 h period [28]. UV radiation is a well-known risk factor for basal cell carcinoma (BCC), and prolonged occupational exposure to sunlight is believed to increase the risk of developing this form of skin cancer. A total of 19 case–control and five cohort studies have reported a significant association between occupational UV exposure and BCC incidence [29].

From a public-health perspective, UV avoidance and photoprotection remain the most actionable radiation-related strategies for cancer risk reduction in the general population.

To provide a concise overview of the most relevant evidence, key preclinical, epidemiological, and occupational studies on carcinogenic exposures such as tobacco, radiofrequency radiation, and ultraviolet radiation, are summarized in Table 1.

### 3.2. Dietary Strategies in Cancer Prevention and Treatment

#### 3.2.1. Intermittent and Periodic Energy Restriction (IF/FMD)

Fasting, traditionally associated with religious practices, involves the intentional avoidance of all food or certain types of food for a specified duration. In oncology, prolonged fasting—lasting several weeks or months—may not be advisable for cancer patients, as it can lead to unintended weight loss, which is often counterproductive. However, shorter-term fasting protocols, implemented over several weeks or months and consisting of fasting periods ranging from 12 to 72 h followed by unrestricted food intake during specific eating windows, may be more feasible and suitable for individuals undergoing cancer treatment [7].

Intermittent fasting (IF), which involves alternating cycles of eating and fasting and typically includes calorie-restricted intake one to three days per week, is attracting growing global interest, particularly in oncology. This is due to its potential to modulate nutrient metabolism and energy homeostasis, support overall health, and possibly influence the development and progression of disease. A related but milder approach is the fasting-mimicking diet (FMD), which entails consuming a low-calorie, low-protein, plant-based diet for three to five days during each chemotherapy cycle [30].

The following sections will explore how nutrition influences cancer-related molecular pathways and how fasting regimens may affect tumor growth and treatment response.

##### Mechanistic Rationale: Cancer-Related Signaling and Nutritional Modulation

Cancer cells display a variety of traits that drive their malignant behavior. These include accelerated growth, impaired or lost tumor suppressor function, the ability to invade and spread to distant tissues and organs, stimulation of new blood vessel formation (angiogenesis), resistance to programmed cell death (apoptosis) and immune surveillance, as well as alterations in metabolic processes. The availability of nutrients such as sugars and amino acids influences several of these hallmarks, partly by modulating key signaling pathways, including insulin-like growth factor-1 (IGF-1), Ras, protein kinase A (PKA), and the PI3K–AKT pathway [31]. Persistent activation of these pathways can also result from mutations in oncogenes. To meet increased energy and biosynthetic needs, these signaling cascades often promote glycolysis, leading to elevated levels of glucose transporters and glycolytic enzymes in cancer cells. As a result, many tumor types become dependent on increased glucose intake or the supply of specific amino acids [32].

Elevated blood glucose levels (hyperglycemia) have been associated with shorter progression-free and overall survival in cancer patients [33]. This underlines the importance of altered metabolic demands and glucose metabolism in cancer progression and patient outcomes, thereby supporting the potential role of nutritional strategies in regulating cancer cell growth and viability. In this context, fasting has demonstrated benefits for long-term health by decelerating the aging process, extending lifespan, reducing inflammation and oxidative damage, promoting cellular regeneration, and enhancing cardiovascular and cognitive functions [34]. Fasting also shows promise as a complementary strategy for cancer prevention and treatment by improving the efficacy and tolerability of anticancer therapies. Additionally, it may improve the quality of life in cancer patients through several adaptive biological responses triggered by the fasting state [35].

##### Fasting Regimens and Tumor Biology

Recent research has shown that altering the energy metabolism of cancer cells can suppress tumor development and enhance the immune system’s capacity to combat tumors. For this purpose, there are several fasting regimes that need to be known; an overview is presented in Table 2.

Intermittent fasting (IF) has been found to decrease the adverse effects associated with chemotherapy and radiotherapy, while simultaneously increasing cancer cells’ sensitivity to these standard treatments. As a result, IF is gaining recognition as a potentially valuable strategy in clinical oncology [36,37].

Implementing fasting or a fasting-mimicking diet (FMD) can lead to reduced levels of blood glucose and insulin-like growth factor-1 (IGF-1). These metabolic changes affect several intracellular signaling pathways, such as PI3K-Akt, Ras, and the mammalian target of rapamycin (mTOR), thereby inhibiting cell growth and proliferation [38]. This is particularly significant because cancer cells are highly dependent on glucose metabolism, which makes them more vulnerable to chemotherapy when FMD is applied. This mechanism is referred to as differential stress sensitization (DSS), and it contributes to enhancing the therapeutic impact of anticancer treatments [43].

Fasting also activates a biological process known as differential stress resistance (DSR), through which healthy cells shift their focus toward protection and internal repair, potentially reducing the damage caused by chemotherapy. Recent preclinical findings suggest that fasting or FMD not only protects normal cells but may also support effector T-cell–mediated cytotoxicity, thereby strengthening the immune response against tumors [39].

Under fasting or FMD conditions, reduced insulin, leptin and IGF-1 levels down-modulate PI3K–Akt, Ras–PKA and mTOR–S6K signaling, decrease glucose transporter (GLUT) activity, and promote a metabolic shift toward oxidative phosphorylation (OxPhos) with increased reactive oxygen species (ROS) generation. These changes activate transcription factors such as FOXO, EGR-1 and p53 in normal tissues, enhancing stress-response and repair pathways that support treatment tolerance [43].

##### Preclinical Evidence on Fasting

The benefits of incorporating fasting into cancer treatment have been strongly supported by preclinical research. Among these are the protection of healthy cells from the harmful side effects of chemotherapy, increased sensitivity of tumor cells to chemotherapy, and enhanced intratumoral infiltration of CD8+ T cells induced by chemotherapy, which collectively contribute to slowing disease progression [44]. In colon cancer mouse models, short-term fasting (STF) causes deficiencies in glucose and amino acids, triggering an anti-Warburg effect. This is marked by increased oxygen consumption without efficient ATP production, leading to elevated oxidative stress and programmed cell death (apoptosis) [45].

Recent mouse studies have demonstrated that intermittent fasting cycles—periods of 12 to 72 h without food followed by refeeding—can have positive impacts on lifespan, age-associated diseases, physiological health markers, stress resistance, metabolic balance, and tissue regeneration [40,46]. In xenograft mouse models of breast cancer, melanoma, and neuroblastoma, combining fasting with high-dose chemotherapy helped protect healthy cells from oxidative stress, DNA damage, and treatment-related endometrial hyperplasia [44]. Remarkably, findings from the same study suggest that two separate 48 h fasting cycles alone were as effective as two rounds of chemotherapy in curbing tumor progression [44].

These effects are thought to result from reductions in blood glucose, insulin, IGF-1, and inflammatory markers, along with increases in IGF-1-binding proteins (IGFBPs) and ketone bodies [47]. Altogether, these findings underscore the potential of non-pharmacological dietary strategies, when used alongside conventional treatments, to significantly improve survival outcomes in certain types of cancer.

##### Clinical and Translational Evidence on Fasting

To date, only a few small-scale clinical trials have explored the impact of various intermittent fasting protocols used alongside chemotherapy, focusing on their effects on cancer progression and prognosis through the evaluation of metabolic and hormonal parameters. These clinical investigations have confirmed that integrating fasting into chemotherapy regimens is both safe and generally well-tolerated. Moreover, fasting may help reduce the toxicity commonly associated with chemotherapy [38,48,49,50].

One clinical study examined the safety, immune-modulating, and metabolic outcomes of a five-day, cyclic fasting-mimicking diet (FMD) administered in combination with standard cancer therapies. In a cohort of 101 patients, the FMD was shown to be feasible and well-tolerated, leading to consistent reductions in blood glucose and growth factor levels. These metabolic shifts resemble those seen in preclinical studies and are believed to contribute to the tumor-suppressive properties of fasting and FMD. Additionally, the FMD alters systemic and tumor-specific immune responses, activating multiple immune mechanisms with antitumor effects [51].

In a broader clinical context, over 300 cancer patients have undergone FMD cycles alongside other therapies, and the occurrence of fasting-related adverse effects has been notably low [48,51,52]. Furthermore, clinical trials have revealed several beneficial outcomes of FMD in terms of cancer-related biological markers, including improved insulin sensitivity, reduced blood glucose, insulin, and leptin levels, and lower systemic inflammation [43].

Ongoing research is now focusing on developing short-term, controlled, and adaptable fasting protocols as cost-effective and powerful adjuncts to conventional cancer treatments. Preclinical models suggest that fasting implemented around chemotherapy or radiation sessions may reduce treatment-related side effects while enhancing therapeutic efficacy [44,45,46,47]. Current human trials aim to evaluate safety, tolerability, and biological mechanisms underlying this promising strategy [48,49,50,51,52]. In underweight or sarcopenic patients, fasting-like regimens should be avoided unless embedded in a supervised protocol with weight and albumin monitoring.

A concise overview of key preclinical and clinical studies on intermittent and periodic energy restriction is provided in Table 3.

#### 3.2.2. Ketogenic Strategies in Oncologic Metabolism (LC/KD)

A ketogenic diet (KD) is characterized by an extremely low carbohydrate (LC) intake and is not primarily designed around calorie restrictions or fasting protocols. Typically, its macronutrient composition includes approximately 90% of caloric intake from fats, while carbohydrates and proteins account for only about 2% and 8%, respectively. These ratios align with the conventional KD formulations, typically represented by a fat-to-carbohydrate ratio of 4:1 and a fat-to-protein ratio of 3:1 [53].

KD is known to effectively stimulate the production of ketone bodies, such as β-hydroxybutyrate and acetoacetate, which can help suppress appetite and maintain low plasma glucose levels in non-cancer individuals [54]. The consistent high intake of fatty acids combined with minimal carbohydrate consumption drives a metabolic shift from glucose-based to fatty acid-based energy production. This increase in fatty acid oxidation leads to elevated concentrations of ketone bodies in the bloodstream [55].

The rationale behind KD in oncology stems from the Warburg effect—a metabolic hallmark of cancer cells where energy is primarily generated through glycolysis and lactate fermentation, even in the presence of oxygen. Cancer cells often exhibit increased glucose uptake to support their rapid growth and proliferation. Therefore, reducing carbohydrate intake through KD can help lower blood glucose, insulin, and IGF-1 levels. As many cancer cells depend on glucose for ATP production, carbohydrate restriction can starve tumor cells while allowing normal cells to rely on ketones for energy [56].

Among ketone bodies, β-hydroxybutyrate has been shown to possess anti-inflammatory effects. It achieves this by downregulating interleukin-1β (IL-1β) expression in bone-marrow-derived macrophages and promoting the expression of mitochondrial uncoupling protein 2, thereby protecting against oxidative stress and supporting increased longevity [57]. Additionally, reduced levels of pro-inflammatory cytokines, such as tumor necrosis factor-α (TNF-α) and interferon-γ (IFN-γ), have been observed with KD, suggesting its potential to enhance cancer treatment outcomes through its anti-inflammatory actions [58].

Evidence also points to KD’s role in hindering colon cancer progression by increasing oxidative stress within tumors. This process suppresses matrix metalloproteinase-9 (MMP-9) expression and promotes the conversion of tumor-associated macrophages (TAMs) from the M2 to the M1 phenotype, both of which are associated with anti-tumor activity [59]. MMPs are crucial contributors to tumor progression, metastasis, angiogenesis, and the degradation of the extracellular matrix [60].

The potential of low-carbohydrate diets like KD in cancer management is currently under investigation, particularly because of cancer cells’ elevated glucose demands compared to normal cells. While preclinical studies in cellular and animal models have shown promising outcomes for KD in cancer prevention and therapy, clinical data on its safety and effectiveness remain limited and preliminary [61].

##### Preclinical Evidence on Ketogenic Diet

Multiple preclinical studies conducted on mouse models have highlighted the antitumor potential of ketogenic diets (KDs) when used as an adjunct to chemotherapy or radiotherapy. These studies have reported significant inhibitory effects against various types of cancer, including colorectal, gastric, prostate, head and neck, brain, and thyroid cancers [61].

In a recent investigation, the effectiveness of KDs in suppressing tumor development was demonstrated in autochthonous animal models of colorectal cancer (CRC). The antineoplastic effects of KDs were primarily attributed to the ketone body β-hydroxybutyrate (BHB), which was shown to inhibit the proliferation of colonic crypt cells and markedly reduce the progression of intestinal tumors. Further analysis suggested that BHB mediates its tumor-suppressive action via activation of the hydroxycarboxylic acid receptor 2 (HCAR2) and the transcriptional regulator homeodomain-only protein homeobox (HOPX), ultimately leading to gene expression changes and suppression of cell growth [62].

Additionally, both BHB and acetone have been identified as modulators of N-methyl-D-aspartate (NMDA) signaling, a mechanism of physiological relevance given the widespread expression of NMDA receptors across various cancer types. Through these mechanisms, the ketogenic diet influences cellular redox balance by altering the ratio between nicotinamide adenine dinucleotide (NAD^+^) and its reduced form (NADH), thereby modulating oxidative phosphorylation (OxPhos) and enhancing the production of reactive oxygen species (ROS) within tumor cells. This redox stress can promote apoptosis and sensitize tumors to cytotoxic therapies, while normal cells are generally protected through improved mitochondrial efficiency and adaptive metabolic responses [63,64].

Collectively, these preclinical findings suggest that ketogenic interventions may exert context-dependent anticancer effects through the regulation of oxidative stress and metabolic signaling pathways involving HCAR2, NMDA, and ROS.

##### Clinical and Translational Evidence on Ketogenic Diet

Several clinical trials have examined the effects of the ketogenic diet (KD) when used alongside chemotherapy, radiotherapy, or antiangiogenic therapies in cancer treatment, producing varied outcomes. Evidence from case reports and small-scale trials suggests that KD is generally safe and well-tolerated by cancer patients. For example, a four-week pilot study involving ten individuals with various types of cancer evaluated the safety and feasibility of KD. The findings showed that patients who experienced stable disease or partial remission had, on average, BHB levels three times higher than those whose disease progressed [65].

Similarly, Schmidt et al. conducted a pilot trial with 16 advanced-stage cancer patients, where 7 participants received a KD supplemented with oil–protein shakes. The study reported a statistically significant average weight reduction without significant changes in blood lipid or cholesterol levels. Some improvements in quality of life were also noted, and no serious adverse events occurred [66].

Another randomized controlled trial involving breast cancer patients evaluated the effects of a medium-chain triglyceride (MCT)-based KD. Results showed reductions in fasting blood glucose, body weight, BMI, and body fat percentage, alongside increased blood ketone concentrations, indicating positive metabolic shifts in the intervention group [67].

Some studies also reported significant reductions in insulin levels among KD participants and an inverse relationship between BHB concentrations and IGF-1 levels [68]. A growing body of literature on adult cancer patients (aged 18 and over) with various malignancies—including glioblastoma, breast, liver, lung, pancreatic, colorectal, and head and neck cancers—has shown encouraging outcomes. These include improved survival, extended progression-free survival, better responses to conventional therapies, and enhanced quality of life [69].

Despite these promising findings, the limited scope and scale of existing studies underscore the need for more extensive research. Larger, controlled trials are necessary to thoroughly evaluate KD’s efficacy in cancer treatment and to explore its potential in combination with other therapeutic strategies [61,64,69]. KD feasibility hinges on adherence and safety checks; cap unintended weight loss (>5% over a cycle) and discontinue if fasting glucose persistently <70 mg/dL or BHB > 4 mmol/L in symptomatic patients [65,66,67].

From a safety standpoint, ketogenic interventions in oncology require careful patient selection and monitoring. Potential adverse effects include gastrointestinal discomfort, dyslipidemia, dehydration, micronutrient deficiencies, and, in some cases, worsening of pre-existing metabolic disorders [64,66,69]. Particular caution is warranted in patients with advanced cachexia, severe hepatic or renal dysfunction, or poorly controlled diabetes, as well as in those at high risk of malnutrition [65,67]. In clinical practice, ketogenic diets should therefore be implemented under the supervision of a multidisciplinary team, with regular assessment of nutritional status, metabolic parameters, and treatment tolerance [68,69].

Representative preclinical and clinical studies on ketogenic strategies in oncology are summarized in Table 4.

#### 3.2.3. Vitamin D, Carotenoids and Signaling Axes

Dietary supplements include ingestible products such as vitamins, minerals, herbal extracts, and other bioactive compounds taken to support health in addition to the usual diet and are distinct from both conventional foods and pharmaceutical drugs [70]. In this section, we focus specifically on vitamin D and carotenoid-related pathways as key micronutrient–signaling axes implicated in cancer risk and progression.

##### 3.2.3.1. Vitamins and Signaling

Vitamin D is among the most extensively investigated micronutrients for its potential role in modulating cancer-related molecular pathways. Over the years, a large body of evidence has examined its capacity to influence carcinogenesis, particularly through effects on cell proliferation, apoptosis, differentiation, and immune regulation [71]. In the liver, both vitamin D2 and D3 are converted into the circulating form 25-hydroxyvitamin D (25(OH)D or calcidiol), which is subsequently transformed in the kidneys into the biologically active hormone 1,25-dihydroxyvitamin D (1,25(OH)_2_D or calcitriol) [70,72,73]. In vitro studies consistently show that vitamin D can suppress tumor growth by promoting apoptosis and reducing proliferative signaling in a variety of cancer cell lines [74]. These mechanistic insights, together with epidemiological data linking sufficient vitamin D status to lower incidence and mortality in several cancer types, have led to growing interest in maintaining adequate 25(OH)D concentrations as part of integrative cancer prevention strategies [75]. Although results vary across populations and tumor sites, current evidence supports a “target-to-sufficiency” approach aimed at preventing deficiency rather than pursuing high-dose supplementation.

Breast Cancer

Vitamin D exerts essential regulatory effects on numerous organs, including the mammary gland, where its actions are mediated through the vitamin D receptor (VDR). Activation of this receptor contributes to limiting uncontrolled cell growth, promoting programmed cell death, and supporting cellular differentiation processes [76]. The early investigations of Abe et al. and Colston et al. were the first to reveal the anti-tumor potential of vitamin D compounds [77,78]. Since then, multiple observational and clinical studies have reinforced the notion that vitamin D status influences both the initiation and progression of breast malignancies [79,80,81]. These observations highlight the relevance of VDR and of enzymes such as CYP27B1 and CYP24A1 in modulating breast cancer (BC) biology [82].

Evidence also indicates that patients diagnosed with triple-negative breast cancer (TNBC) often display the lowest serum vitamin D levels among breast cancer subtypes, suggesting a possible protective role of adequate vitamin D exposure in this population [82]. In addition, a meta-analysis by Bauer et al. including 5206 BC cases and 6450 controls found a nonlinear inverse association between circulating vitamin D concentrations and breast cancer risk in postmenopausal women [82,83]. Experimental findings from cell and animal models further show that 1,25(OH)_2_D_3_ and related analogues regulate a wide spectrum of cancer-relevant pathways—including proliferation, apoptosis, differentiation, epithelial–mesenchymal transition, autophagy, metabolic activity, and cancer stem cell populations—within breast tissue [84].

Colorectal Cancer

Vitamin D has also been the focus of extensive research for its potential role in lowering colorectal cancer (CRC) risk [71]. Higher dietary or supplemental intake of vitamin D appears to correlate with reduced likelihood of early-onset CRC and precursor lesions. Several mechanisms have been proposed to explain this association, such as vitamin D–mediated inhibition of cell proliferation, reduced migratory and invasive capacity, suppression of angiogenesis in malignant colon cells, and immune-modulating effects on intestinal lymphocytes and macrophages [85]. Moreover, higher pre-diagnostic serum concentrations of 25-hydroxyvitamin D [25(OH)D] are associated with improved overall and colorectal cancer-specific survival, with pooled hazard ratios of 0.68 and 0.67 for the highest versus lowest categories [86].

Findings from the COLON cohort further suggest that patients with 25(OH)D concentrations above 50 nmol/L, together with magnesium intakes exceeding the population median, experience reduced mortality risk [87,88]. In a randomized trial by Ng et al., serum 25(OH)D levels were evaluated in 515 individuals undergoing chemotherapy for stage IV CRC, revealing a high prevalence of vitamin D insufficiency (<20–30 ng/mL) in this group [89]. A large cohort analysis by Yuan et al., involving 1041 patients with metastatic or advanced CRC, confirmed widespread deficiency of vitamin D within this clinical population [90].

Lung Cancer

Altered vitamin D status and disruptions in VDR signaling have been implicated in the development of several malignancies, including lung cancer [74]. Epidemiological work indicates that maintaining adequate vitamin D levels may help reduce lung cancer risk, with multiple studies reporting an inverse association between serum vitamin D and lung cancer incidence [74]. Higher circulating vitamin D concentrations have also been linked to improved survival and lower mortality among lung cancer patients [91]. Supporting this, a Finnish cohort study showed that vitamin D insufficiency was associated with greater lung cancer risk, particularly among women and younger adults [74,92].

Genetic variability within vitamin D pathway components may additionally affect individual susceptibility to lung cancer and influence therapeutic responses [74,93]. In vitro research demonstrates that vitamin D can increase expression of the tumor-suppressor gene p53 while diminishing the expression of the anti-apoptotic gene Bcl-2 in A549 lung carcinoma cells [74,93]. Vitamin D has also been shown to enhance VDR-mediated repression of the epidermal growth factor receptor (EGFR) gene, suggesting that vitamin D/VDR signaling could represent a therapeutic target for EGFR-driven lung cancers [74,94,95].

Ovarian Cancer

A substantial body of research suggests that vitamin D may offer protective effects against ovarian cancer. Data from experimental models, including cell culture systems, human xenografts, and mouse assays, indicate that vitamin D can reduce tumor cell proliferation, enhance apoptosis, and slow tumor progression. Mendelian randomization studies further support a causal relationship, showing that genetically lower concentrations of 25(OH)D are associated with higher ovarian cancer risk [72,96].

An Australian cohort found that women with higher serum 25D_3_ concentrations at diagnosis experienced longer survival after invasive ovarian cancer [97]. Similar results have been observed in European populations, where genetically reduced 25D_3_ levels were linked to increased disease risk [98]. Genome-wide association studies (GWAS) likewise point toward a protective role for vitamin D, suggesting that higher circulating levels may reduce the likelihood of epithelial ovarian cancer (EOC) development [99].

A case–control study involving 1631 women with EOC demonstrated that elevated 25(OH)D levels were associated with improved survival outcomes [100]. Additional investigations examining polymorphisms in the VDR gene suggest that genetic variation may contribute to both ovarian cancer susceptibility and survival probability, by altering vitamin D signaling efficiency [101,102,103,104,105]. Future research integrating genomic, epigenomic, proteomic, transcriptomic, and metabolomic data will be essential for optimizing vitamin D–based preventive or therapeutic approaches and for identifying subgroups most likely to benefit [71,106].

##### 3.2.3.2. Carotenoids and Retinoid-Related Pathways

Carotenoids—pigments naturally occurring in fruit and vegetable sources—are widely recognized for their antioxidant, immune-modulating, and anti-mutagenic properties. A large body of research has linked higher dietary intake of specific carotenoids to a lower risk of several cancer types. Although such associations do not demonstrate direct causality, they consistently point toward a potential protective effect against carcinogenesis. Beyond their coloration functions, carotenoids serve as precursors of essential biomolecules such as vitamin A and contribute to various antioxidant defense processes [107,108].

Through their capacity to neutralize reactive oxygen species (ROS), carotenoids may counteract oxidative damage implicated in the initiation of cancer, neurodegenerative or cardiovascular diseases, and age-related conditions. Experimental studies also show that carotenoids can influence key cellular events—including apoptosis, gene transcription, angiogenesis, and immune activities—highlighting their broad biological relevance [108,109]. Moreover, habitual consumption of carotenoid-rich foods appears to modulate intracellular pathways tied to antioxidant protection [110].

Head and Neck Cancer

In cancers of the oral cavity, pharynx, and larynx collectively defined as head and neck cancer (HNC), carotenoid intake has been repeatedly associated with reduced disease risk, likely due to their roles in mitigating oxidative stress, DNA damage, and immune dysregulation. Although the mechanistic details remain incompletely understood, multiple investigations suggest that carotenoids exert a measurable protective effect [109].

Two meta-analyses by Leoncini et al. evaluated dietary carotenoid exposure across sixteen epidemiological studies, demonstrating that β-carotene intake correlated with a 46% reduction in oropharyngeal cancer (OPC) risk and a 57% reduction in laryngeal cancer (LC) [107,111]. Likewise, β-cryptoxanthin and lycopene were associated with risk reductions of 59% and 50% for LC, respectively. Intake of α-carotene, β-cryptoxanthin, and lycopene was further linked to a 26% lower risk of oral cavity (OCC) and pharyngeal (PC) malignancies [107,109].

A pooled re-analysis of ten case–control studies conducted across North America, Europe, and Japan involving 18,207 cases and 12,248 controls similarly found that individuals with the highest carotenoid consumption had approximately a 39% lower likelihood of OCC, PC, and LC [111].

Breast Cancer

A growing body of literature indicates that carotenoids may contribute to lowering breast cancer risk. In vitro evidence shows that β-carotene, at concentrations around 1 μM, downregulates anti-apoptotic proteins such as PARP and Bcl-2. Lycopene, at doses ≥ 2 μM, has been shown to suppress proliferation in MCF-7 breast cancer cells while enhancing pro-apoptotic signaling via increased p53 and Bax expression [112,113].

Fulan Hu and colleagues, in a dose–response meta-analysis, reported that higher dietary intake of α-carotene was significantly associated with reduced breast cancer risk, although conclusions regarding β-carotene remain inconsistent and warrant further clarification [114].

A large investigation involving over 12,000 women (5707 cases, 6389 controls) across several U.S. states found that greater vegetable, fruit, and carotenoid intake was linked to reduced breast cancer risk in premenopausal women. This association was not observed among postmenopausal women, particularly those who smoked [115]. Collectively, the evidence supports the biological capacity of carotenoids to limit breast cancer cell growth [112,113].

Colorectal Cancer

Epidemiological and clinical investigations have identified significant associations between colorectal cancer (CRC) risk and circulating or dietary carotenoid levels. In vitro, lycopene at concentrations ≥ 2 μM reduces proliferation of HT-29 colorectal cancer cells, likely through inhibition of the PI3K/Akt signaling cascade [116]. Animal studies further show that lycopene elevates p21 and PCNA expression while dampening inflammatory mediators such as PGE2, COX-2, and phosphorylated ERK1/2. Additionally, plasma MMP-9—an enzyme involved in invasion and metastasis—correlates inversely with lycopene intake [117].

A meta-analysis by Han et al., evaluating 22 studies, confirmed that higher concentrations of β-cryptoxanthin, α-carotene, β-carotene, and lycopene are consistently associated with reduced CRC risk, reinforcing the hypothesis that carotenoids may inhibit carcinogenic progression in the colon [118].

Lung Cancer

Carotenoid intake appears to modulate lung cancer risk, with several studies demonstrating a clear dose–response gradient. Each additional milligram of dietary β-carotene per day has been linked to an approximate 2% decrease in lung cancer risk, while 10 μg/day increments of β-cryptoxanthin correspond to a 1% reduction. Lycopene intake shows a similar pattern, with an estimated 3% decreased risk per additional milligram consumed daily [119].

A Hawaiian case–control study reinforced these findings, reporting lower lung cancer incidence among individuals consuming carotenoid-rich vegetables [120]. A comprehensive meta-analysis by Gallicchio et al.—which included both clinical trials and observational data—also documented an overall inverse association, though the magnitude of risk reduction was modest and did not consistently reach statistical significance [119]. Importantly, β-carotene supplementation (as pills) has demonstrated harmful effects in smokers, emphasizing that benefits relate to food-based carotenoid sources rather than high-dose isolated supplements.

Prostate Cancer

Carotenoids such as α-carotene, β-carotene, and particularly lycopene have been extensively examined in relation to prostate cancer. More than seventy studies have explored these associations, with most identifying an inverse relationship between lycopene exposure—through diet or serum levels—and prostate cancer risk. In cell culture models, lycopene significantly impairs proliferation and disrupts normal cell cycle progression in prostate cancer lines [112,121].

A nested case–control study evaluating cis-lycopene, trans-lutein, β-trans-carotene, and dihydrolycopene reported that trans β-carotene isomers were associated with increased prostate cancer risk among African American men, irrespective of smoking status or multivitamin use [122].

Another case–control investigation involving 65 prostate cancer patients and 132 matched controls found that higher plasma levels of lycopene and zeaxanthin were linked to reduced cancer risk, with β-cryptoxanthin and lutein showing borderline protective associations [123].

It should be emphasized that much of the evidence linking vitamin D status and carotenoid intake with cancer risk and outcomes is derived from observational cohorts and case–control studies. Although these data consistently support inverse associations for several malignancies, they are subject to residual confounding and cannot establish causality. Randomized trials and well-designed intervention studies remain comparatively sparse, and future research is needed to clarify dose–response relationships, optimal target levels, and potential subgroup-specific effects.

Representative observational and interventional studies on vitamin D and carotenoids in relation to cancer risk and outcomes are summarized in Table 5.

#### 3.2.4. Cancer-Specific Evidence Summary for Dietary Strategies

Evidence for dietary strategies varies substantially across cancer types. Fasting and fasting-mimicking diets (FMD) show the strongest and most coherent preclinical and early clinical effects in breast and colorectal cancers, demonstrating reduced chemotherapy toxicity, improved metabolic responses, and enhanced antitumor immunity [44,45,48,49,50,51,52]. Ketogenic interventions, while supported by robust mechanistic and preclinical data, have been investigated clinically mainly in glioblastoma, breast, and pancreatic cancers, with small but promising trials indicating feasibility and metabolic benefits [61,62,63,64,65,66,67,68,69].

Associations between vitamin D and carotenoids and cancer risk are best documented for breast, colorectal, and lung cancers, supported by epidemiological studies, meta-analyses, and mechanistic evidence linking these micronutrients to cell proliferation, apoptosis, immune modulation, and signaling pathways [71,72,73,74,75,76,77,78,79,80,81,82,83,84,85,86,87,88,89,90,91,92,93,107,108,109,110,111,112,113,114,115,116,117,118,119,120,121,122]. Epidemiological analyses suggest that maintaining vitamin D sufficiency may contribute to lower incidence and improved survival across several tumor sites, supporting a “target-to-sufficiency” rather than high-dose supplementation approach [75]. For other malignancies, including ovarian, prostate, and head-and-neck cancers, evidence remains heterogeneous and often relies on observational data; therefore, clinical translation should be approached with caution [86,87,88,89,90,96,97,98,99,100,101,102,103,104,105,109,110,111,122,123].

### 3.3. Physical Activity, Sedentary Behavior, and Exercise Oncology

At present, smoking remains the leading modifiable risk factor for cancer. However, physical inactivity is rapidly emerging as the second-most significant preventable contributor to cancer development [124]. According to the World Health Organization (WHO), more than 1.4 billion adults—over a quarter of the global population—do not engage in sufficient physical activity [125]. With obesity now affecting individuals at younger ages, people are increasingly exposed to related health risks throughout their lives. High-income countries report rates of physical inactivity that are approximately twice as high as those in low-income nations. The global trend toward reduced physical activity has been steadily rising, posing negative consequences for environmental sustainability, economic productivity, quality of life, and the overall health of communities [126,127].

Regular engagement in physical activity is widely recognized as a key preventive measure against non-communicable diseases such as cardiovascular disease, hypertension, type 2 diabetes, stroke, and numerous types of cancer [6,128,129,130,131,132,133,134,135]. For decades, epidemiological research has consistently linked higher levels of physical activity with a lower risk of several common cancers. In 2021, the global burden of cancer attributable to elevated body mass index (BMI) was estimated at approximately 356,700 deaths and 8.89 million disability-adjusted life years (DALYs), representing a substantial increase compared to previous decades [136].

#### 3.3.1. Prevention: Physical Activity and Cancer Risk

Any bodily movement driven by skeletal muscles that leads to energy expenditure qualifies as physical activity. While exercise is a subset of physical activity, it encompasses a wide range of intensities and forms, including occupational tasks, household chores, commuting, and recreational pursuits like walking, cycling, sports, and active play [137]. Engaging in physical activity positively influences the body’s metabolic processes, making it more resistant to various types of cancer. The nature and intensity of the activity can impact metabolism and energy balance, thereby influencing how much food and nutrients can be consumed without leading to fat accumulation. When caloric intake exceeds energy expenditure, it results in a positive energy balance, promoting weight gain and increased body fatness [137].

Although physical activity is often emphasized for weight management, it also offers direct cancer-protective benefits through several biological mechanisms. These include modulation of the immune system [138,139,140], reduction in circulating estrogen [141], decreased oxidative stress [142], improved insulin sensitivity [143], suppression of inflammation [144], favorable epigenetic changes [145], reduced telomere shortening [146], and enhanced DNA repair [139]—all of which are associated with the initiation and progression of cancer.

Independent of its effects on obesity, physical activity has been inversely associated with colon and breast cancer, as demonstrated in a 2020 Mendelian randomization study using UK Biobank data. This inverse relationship was found to be independent of BMI [147].

Moreover, strength training, which builds skeletal muscle mass, is linked to lower risks of kidney, bladder, and colorectal cancers [148,149]. Aerobic exercise also improves glucose metabolism and insulin sensitivity, potentially reducing the risk of cancers associated with insulin resistance, such as colon, liver, pancreatic, and endometrial cancers [150,151]. While not all cancers are equally affected by physical activity, lifestyle interventions, including regular exercise, have shown potential to lower overall cancer risk [152]. For example, intense domestic activities like heavy housework may offer protective benefits against breast cancer. Exercising for three to five hours per week has been associated with a reduced risk of developing cancer [153].

One study found that individuals who exercised had a 15–20% lower risk of breast cancer and a 24% reduced risk of colorectal cancer [154]. Exercise has been shown to reduce the risk of breast cancer in postmenopausal women and may help protect them from autonomic nervous system dysfunction [155,156]. Numerous clinical trials and meta-analyses have demonstrated that exercise significantly reduces the risk of colorectal cancer, supporting its role as an effective preventive strategy [157]. Similarly, a meta-analysis on stomach cancer, encompassing 10 cohort and 12 case–control studies, reported a 19% risk reduction among those who exercised more frequently [158].

Given that obesity is a known risk factor for endometrial cancer, exercise-induced weight loss plays a protective role. According to a review of 33 studies, women who exercised regularly had a 20% lower risk of endometrial cancer [159]. A similar review on esophageal cancer found that those with the highest levels of physical activity had a 21% lower risk compared to the least active individuals [160]. In a large-scale study involving over 1 million participants, leisure-time exercise was associated with a 13% lower risk of bladder cancer and a 23% lower risk of kidney cancer [161]. A review of 25 studies found that while physical activity had little effect on lung cancer risk in non-smokers, it did lower the risk among smokers [162].

#### 3.3.2. Physical Activity During and After Therapy

Beyond its role in cancer prevention, exercise has been shown to reduce the adverse effects associated with cancer treatments. Therapies such as chemotherapy and targeted treatments often produce side effects including fatigue, cognitive decline, depression, muscle and bone loss, and cardiac toxicity. These side effects can negatively impact treatment outcomes and overall quality of life [152]. In some cases, patients are unable to complete or tolerate therapy due to the severity of these side effects, potentially altering the progression of the disease. However, substantial evidence indicates that engaging in physical activity can help alleviate many of these treatment-related complications.

Exercise training influences both cancer-specific and physiological as well as psychological outcomes associated with treatment [128,129,130,131,132]. Not only can exercise reduce the side effects of therapy, but it may also enhance treatment efficacy, making it a valuable addition to the therapeutic process [163]. While some healthcare professionals express concern about whether physical activity could influence survival or prognosis, current research suggests that exercise may not significantly affect survival rates for cancers such as gastric cancer, non-Hodgkin lymphoma, and several others [164].

Exercise can also be leveraged as a form of cancer-suppressive therapy by disrupting cancer metabolism, particularly anaerobic glycolysis. The impact of physical activity on tumor metabolism depends on its frequency, duration, intensity, and type. By decreasing vasoconstriction in hypoxic environments, exercise improves tumor perfusion and reduces hypoxia, making the cancer microenvironment less aggressive and more susceptible to treatment [165,166].

Current consensus statements support prescribing at least 150 min per week of moderate-intensity aerobic activity, combined with 2–3 weekly sessions of resistance training, with appropriate adaptations based on treatment phase, symptom burden, and comorbidities. These recommendations align with the growing field of exercise oncology, which emphasizes structured, individualized training programs to optimize treatment tolerance and quality of life [126,137,164].

Additionally, regular physical activity helps counteract immunosenescence, the aging of the immune system, which is marked by decreased natural killer (NK) cell activity, chronic inflammation, impaired antigen presentation by dendritic and monocyte cells, and a reduction in naïve T cells capable of targeting emerging cancer cells. These changes contribute to the heightened cancer risk in older adults [167]. Exercise has been shown to slow immune aging by enhancing immune cell function [145], thereby playing a protective role against immune-related malignancies.

In summary, robust evidence supports that physical activity reduces cancer risk, curbs tumor growth and progression, mitigates the side effects of cancer treatments, improves treatment adherence, and enhances patient quality of life. A pragmatic mix of aerobics plus resistance exercise counters treatment-related sarcopenia and supports dose intensity, even when total weekly volume is modest.

#### 3.3.3. Adiposity and Metabolic Health

A body with overweight or obesity (defined as excessive fat accumulation) contains a higher proportion of fat compared to lean tissues such as muscle and bone. Carrying excess weight at unhealthy levels increases the risk of developing certain types of cancer and raises the likelihood of recurrence after treatment. According to the World Health Organization, in 2016, over 340 million children and adolescents and 1.9 billion adults were living with overweight or obesity, a number projected to rise further [168]. The global cancer burden is expected to continue growing due to ongoing trends of reduced physical activity and increased body fat, particularly in the context of an aging global population. If these trends persist, overweight and obesity may soon surpass smoking as the leading preventable cause of cancer.

##### Obesity and Cancer: Epidemiology and Mechanisms

Since the early 20th century, the relationship between body fat and cancer has been explored in scientific literature, with more focused discussions emerging since 1987 [169]. As the prevalence of overweight and obesity continues to rise, so does the incidence of cancers associated with excess body fat. This trend is expected to significantly increase the cost of future cancer treatments and place a growing financial burden on healthcare systems due to the long-term management of related comorbidities.

Maintaining a healthy weight is one of the most effective strategies for cancer prevention. Importantly, excess body fat during childhood has been linked to an increased risk of developing various diseases, including cancer, later in life. Hidayat et al. identified associations between early-life adiposity and the development of eight different types of cancer in adulthood [170]. Moreover, weight gain in adulthood is associated with a higher risk of overweight, obesity, and postmenopausal breast cancer, with a 6% increased risk for every 5 kg gained [171]. Despite growing evidence of the link between obesity and cancer in adults, the impact of weight gain throughout adulthood is often underestimated.

Body fatness contributes to cancer risk through several biological mechanisms. Obesity is associated with elevated fasting insulin levels and increased inflammatory mediators, both of which promote cell proliferation (Hallmark: sustained proliferative signaling) [172]. Additionally, obesity can impair apoptosis, the process by which damaged cells are eliminated, thereby allowing abnormal cells to survive (Hallmark: resisting cell death) [173]. Cancer cells can also spread through the bloodstream or lymphatic system (Hallmark: activating invasion and metastasis). These endocrine and metabolic disruptions, particularly involving insulin and inflammation, highlight the complex relationship between obesity and cancer development.

##### Clinical Weight-Loss Strategies in Oncology

Combined dietary energy restriction and progressive exercise represent first-line, scalable interventions to reduce adiposity, improve insulin resistance, and lower low-grade inflammation, mechanisms tightly linked to carcinogenesis and poorer outcomes [6,143,144]. Structured programs should favor protein-adequate, minimally processed diets and supervised training tailored to treatment phase and fatigue [128].

##### Survivorship and Relapse Risk

Post-treatment weight gain is common and associated with higher recurrence and mortality in several cancers [152,171]. Survivorship care plans should embed long-term weight management with behavioral support, periodic monitoring, and referral pathways to exercise oncology services [152].

A concise overview of representative epidemiological and interventional studies on physical activity, adiposity, and cancer outcomes is provided in Table 6.

#### 3.3.4. Cancer-Specific Evidence Summary for Physical Activity, Sedentary Behavior, and Adiposity

Evidence consistently shows that physical activity lowers the risk of several major cancers, with the strongest and most reproducible reductions observed in breast, colorectal, and prostate cancers [139,147,157]. Protective effects for endometrial, kidney, esophageal, and gastric cancers are also supported by epidemiological and meta-analytic data [158,159,160,161]. For lung and pancreatic cancers, activity appears beneficial mainly among high-risk individuals (e.g., smokers), though results remain heterogeneous [139,162].

In contrast, adiposity and metabolic dysfunction increase cancer risk across multiple sites. Excess body fatness contributes to carcinogenesis through hyperinsulinemia, inflammation, and impaired apoptosis [145,172,173], and is strongly linked to postmenopausal breast, colorectal, endometrial, kidney, liver, and esophageal adenocarcinoma risk [136,159,160,161,171]. Weight gain during survivorship further worsens recurrence and mortality in several cancers [152,171].

Overall, cancer-specific patterns highlight the need for integrated strategies combining regular physical activity, reduced sedentary time, and evidence-based weight management to lower incidence and improve outcomes across tumor types.

### 3.4. Complementary and Mind–Body Approaches

The term complementary and alternative medicine (CAM) refers to a wide array of medical and health-related practices and products that fall outside the scope of conventional medicine. Among individuals with cancer, CAM is often used alongside standard treatments to help alleviate stress and anxiety related to the disease and its therapies, as well as to manage side effects such as nausea, pain, and fatigue. Common CAM approaches in oncology include herbal medicine, acupuncture, mind–body practices—such as meditation, yoga, and Tai Chi—nutritional supplements, energy-based therapies, and massage [8,9,10,11].

The use of CAM among cancer patients has been steadily increasing. It is estimated that between 33% and 47% of patients worldwide incorporate complementary, alternative, or integrative therapies into their care [12]. One of the most burdensome symptoms experienced by cancer patients is cancer-related fatigue (CRF), which significantly impairs quality of life by affecting daily functioning, social engagement, work performance, leisure activities, and interpersonal relationships. CRF can also hinder adherence to prescribed cancer treatments.

As a result, many patients turn to CAM in an effort to regain a sense of well-being and better manage their symptoms [13,14]. While some of these therapies may offer symptom relief and contribute to improved quality of life, their therapeutic efficacy remains variable. Further research is needed to determine how best to integrate these modalities into evidence-based cancer care. Given heterogeneity in quality and potential drug–herb interactions, CAM use should be discussed with the oncology team and, where possible, evaluated within clinical protocols.

Given the potential for herb–drug interactions and the variable quality of over-the-counter products, complementary therapies, particularly herbal supplements, should be used only under the supervision of qualified healthcare professionals [8,9,10,11,12]. Several botanicals commonly used by cancer patients, such as St. John’s wort (*Hypericum perforatum*), ginseng, and high-dose curcumin extracts, can alter the metabolism of chemotherapeutic agents through cytochrome P450 modulation or effects on P-glycoprotein transporters [13,14]. Therefore, CAM interventions should be discussed with the oncology team and incorporated into care plans only when clinically appropriate.

A concise summary of representative clinical and observational studies examining complementary and mind–body approaches in oncology is provided in Table 7. This table highlights the most commonly investigated interventions, cancer types studied, and the primary outcomes reported across the literature.

#### Cancer-Specific Evidence Summary for Complementary and Mind–Body Interventions

The effectiveness of complementary and mind–body interventions varies significantly across cancer types. The strongest and most consistent evidence is available for breast and gynecological cancers, where acupuncture, yoga, and meditation have demonstrated benefits in reducing treatment-related fatigue, anxiety, pain, and overall symptom burden [8,9,10,11]. In prostate and hematologic cancer survivors, practices such as Tai Chi, Qigong, and yoga show promising improvements in fatigue, sleep quality, and functional capacity, although the evidence base remains smaller [12,13]. Acupuncture has been most extensively studied in breast cancer populations [9,10], while evidence for digestive, thoracic, and other solid tumors is still limited and largely preliminary [13,14]. Overall, the variability in response patterns across tumor sites highlights the need for more targeted, tumor-specific trials in integrative oncology. Given the heterogeneity in study quality and the potential for herb–drug interactions associated with several complementary therapies, cancer-specific recommendations should be individualized and implemented under the supervision of qualified healthcare professionals.

## 4. Limitations

Since this is a narrative review, a systematic study selection protocol was not followed (no protocol registration, no predefined eligibility with dual independent screening, and no formal risk-of-bias assessment). This may introduce a selection bias, as the inclusion of articles was partially based on the authors’ criteria. However, this approach allowed for a deeper and more critical discussion of the available evidence, facilitating the formulation of practical recommendations based on the most relevant literature. Nonetheless, the non-systematic search strategy may have led to the omission of relevant studies, particularly those not indexed in the selected databases and to susceptibility to publication bias. The English-language focus may also have excluded pertinent non-English studies, and heterogeneity across study designs and populations limits generalizability. Some emerging or less-studied interventions may have been underrepresented due to the broad scope of the review. Accordingly, the findings should be interpreted as hypothesis-generating rather than causal. Future systematic reviews and meta-analyses are warranted to validate and expand upon the insights presented here.

## 5. Conclusions and Future Perspectives

Modifiable lifestyle factors—including reducing exposure to carcinogens (tobacco and ultraviolet radiation), optimizing dietary patterns, maintaining a healthy body weight, and engaging in regular physical activity—play a decisive role in cancer prevention and management. A growing body of epidemiological and mechanistic evidence confirms that adopting healthy behaviors significantly reduces cancer incidence, improves treatment tolerability, and enhances long-term survivorship outcomes.

Exposure to well-established carcinogens such as tobacco smoke and ultraviolet radiation remains a leading preventable cause of cancer worldwide. Strengthening tobacco-cessation strategies and promoting photoprotection continue to represent essential public-health priorities.

Dietary strategies—including intermittent and periodic energy restriction, fasting-mimicking diets, ketogenic protocols, and micronutrient-related pathways such as vitamin D and carotenoids—show promise in modulating tumor metabolism and improving treatment tolerance, although evidence varies considerably by cancer type and study design. Future research should clarify optimal protocols, tumor-specific indications, and long-term safety.

Regular physical activity emerges as a key modulator of cancer risk and prognosis by influencing hormonal regulation, immune function, oxidative stress, and inflammation. Excess adiposity, increasingly prevalent across all age groups, is strongly associated with higher cancer risk and poorer post-diagnosis outcomes. The obesity epidemic, driven by sedentary lifestyles and energy-dense diets, poses a major public-health challenge that may soon surpass tobacco as the leading preventable cause of cancer. Weight management through a combination of structured exercise and targeted nutritional interventions should therefore be prioritized in cancer-control strategies.

In addition to conventional medical approaches, the integration of evidence-based complementary and mind–body therapies into oncology care is gaining traction. Modalities such as meditation, yoga, Tai Chi, acupuncture, and selected herbal preparations may help manage symptoms like fatigue and anxiety, provided they are used under appropriate clinical supervision due to potential herb–drug interactions. Rigorous clinical and translational studies remain essential to define their safety profiles, mechanisms of action, and clinical applicability.

From a clinical and public-health standpoint, lifestyle interventions remain the most accessible, cost-effective, and impactful tools for cancer prevention and control. Future research should refine individualized prevention strategies, define dose–response relationships for diet and physical activity, and explore the interplay between lifestyle, tumor biology, and genetic predisposition. Moreover, creating supportive environments through urban planning that promotes active living and community-based health education will be critical to sustaining behavioral change across the lifespan.

Ultimately, lifestyle medicine should be regarded as a core pillar of modern oncology, integrating nutrition, exercise, carcinogen reduction, and behavioral interventions within standard care pathways. Implementation priorities include embedding exercise oncology and nutrition services in cancer centers, developing pragmatic home- and community-based programs, and ensuring equitable access across healthcare systems.

## Figures and Tables

**Table 1:** Representative studies on carcinogenic exposures: tobacco, radiofrequency electromagnetic fields (RF-EMF), and ultraviolet radiation (UV).

Author, Year [Ref]	Design/Sample	Exposure Type	Main Findings
**Li & Hecht, 2022** [18]	Comprehensive toxicological review	Tobacco smoke constituents	Identified > 79 carcinogens in tobacco; mechanistic evidence linking nitrosamines, PAHs, VOCs, cadmium to multiple cancers.
**Larsson et al., 2020** [19]	Mendelian randomization (UK Biobank + consortia)	Smoking, alcohol	Strong causal association with lung, bladder, kidney, GI, pancreatic, liver cancers.
**Secretan et al., 2009** [20]	Systematic review (IARC)	Tobacco, environmental exposures	Classified tobacco and tobacco smoke as Group 1 carcinogens for multiple organs.
**Wei et al., 2009** [21]	In vitro study	Tobacco-specific NNK carcinogen	Increased colon cancer cell migration via α7-nAChR; mechanistic evidence for metastasis.
**Hecht, 2002** [22]	Mechanistic review + human biomarker evidence	Tobacco-derived carcinogens	Demonstrated transfer and metabolic activation of PAHs in breast tissue of smokers.
**Jethwa & Khariwala, 2017** [23]	Review of clinical epidemiology	Tobacco (alone or with alcohol)	70–80% of HNSCC attributable to tobacco/alcohol; ~10× increased risk vs. non-smokers.
**Caliri et al., 2021** [24]	Mechanistic review	Tobacco oxidative stress	Showed ROS/RNS-driven macromolecular damage as a central mechanism of carcinogenesis.
**Stang et al., 2001** [26]	Case–control (hospital + population)	RF-EMF occupational exposure	Increased risk of uveal melanoma in RF-exposed workers (radios, mobile devices).
**Karipidis et al., 2007** [27]	Case–control (Australia)	Occupational RF exposure	Potential increase in glioma risk among RF-exposed workers.
**Gupta et al., 2022** [28]	Review (ICNIRP guidelines)	Non-ionizing radiation, UV	Summarized safety limits and carcinogenic potential of artificial UV exposure.
**Bauer et al., 2011** [29]	Systematic review + meta-analysis	Occupational UV exposure	Strong association between solar UV exposure and basal cell carcinoma (BCC); 19 case–control + 5 cohort studies.

Notes: This table summarizes representative toxicological, mechanistic, epidemiological, and occupational studies on major carcinogenic exposures relevant to cancer risk. Abbreviations: RF-EMF = Radiofrequency electromagnetic fields; UV = Ultraviolet radiation; PAHs = Polycyclic aromatic hydrocarbons; VOCs = Volatile organic compounds; NNK = Nicotine-derived nitrosamine ketone; HNSCC = Head and neck squamous cell carcinoma; BCC = Basal cell carcinoma; ROS/RNS = Reactive oxygen/nitrogen species; IARC = International Agency for Research on Cancer.

**Table 2:** Overview of fasting-related dietary regimens.

Author & Year [Ref]	Fasting Regimen	Definition
**Patterson & Sears, 2017** [36]	Fasting	Complete caloric abstention or exclusion of selected foods for a defined interval
**Patterson & Sears, 2017** [36]**; Longo & Mattson, 2014** [37]**; Safdie et al., 2009** [38]**; Brandhorst, 2021** [39]	Intermittent fasting	Alternation of eating and energy-restriction or water-only intervals on 1–3 days per week
**Longo et al., 2021** [40]	Periodic fasting	Severe energy restriction or water-only phases lasting ~48 h (up to 1 week in some protocols)
**Turbitt et al., 2019** [41]	Short-term fasting	Time-limited abstention (≈12–72 h), e.g., alternate-day patterns
**Brandhorst, 2021** [39]**;** **Wei et al., 2019** [42]	Fasting-mimicking diet	Brief, hypocaloric, low-protein, plant-forward cycles aligned to treatment sessions

**Table 3:** Summary of key preclinical and clinical studies on intermittent and periodic energy restriction (IF/FMD) in oncology.

Author & Year [Ref]	Study Design	Tumor Type	Sample Size	Intervention (IF/FMD Protocol)	Main Outcomes
**Bianchi et al., 2015** [45]	Preclinical (colon cancer models)	Colorectal cancer	n/a	Short-term fasting (STF)	Anti-Warburg shift, ↑ ROS, ↑ apoptosis, ↓ tumor growth.
**Lee et al., 2012** [44]	Preclinical (mouse xenograft)	Breast, melanoma, neuroblastoma	n/a	48 h fasting cycles + chemotherapy	Sensitization to chemotherapy; DSR/DSS; slowed tumor growth.
**Turbitt et al., 2019** [41]	Preclinical/translational review	Immunotherapy-relevant tumors	n/a	IF + CR mimetics	Enhanced antitumor immunity and immunotherapy response.
**Longo et al., 2021** [40]	Preclinical review	Multiple models	n/a	Periodic fasting	Improved stress resistance, metabolic remodeling, immune activation.
**Safdie et al., 2009** [38]	Case series	Mixed solid tumors	10	48–72 h fasting	Safe; reduced chemotherapy-related side effects.
**de Groot et al., 2015** [48]	Randomized pilot trial	Breast cancer (HER2–)	26	24–48 h fasting pre-chemotherapy	↓ DNA damage; improved treatment tolerance.
**Dorff et al., 2016** [49]	Pilot clinical trial	Various solid tumors	20	24–72 h fasting cycles	Safe; reduced fatigue; metabolic modulation observed.
**Bauersfeld et al., 2018** [50]	Randomized cross-over trial	Breast & ovarian cancer	34	Short-term fasting (48–72 h)	Improved QoL; fewer side effects.
**Wei et al., 2019** [42]	Clinical metabolic study	Mixed populations	>100	Cyclic 5-day FMD	↓ IGF-1, ↓ glucose, ↓ CRP.
**de Groot et al., 2020** [52]	Multicenter randomized Phase II trial (DIRECT)	Breast cancer	131	FMD during neoadjuvant chemotherapy	↓ toxicity; favorable metabolic responses.
**Vernieri et al., 2022** [51]	Translational clinical trial	Multiple solid tumors	101	5-day cyclic FMD	Safe; metabolic & immunologic remodeling.
**Brandhorst, 2021** [39]	Clinical/translational review	Chemotherapy augmentation	n/a	FMD cycles around chemotherapy	Improved treatment tolerance; favorable immunometabolic changes.

Notes: This table summarizes representative preclinical and clinical studies investigating intermittent fasting (IF), short-term fasting (STF), periodic fasting (PF), and fasting-mimicking diets (FMD) in the context of cancer biology, treatment tolerance, and metabolic/immunologic modulation. Sample sizes are reported only for human studies when applicable. Preclinical findings refer to murine or cell-based models as indicated by the original authors. “Outcomes” refer to the primary antitumor or metabolic effects described in each study. Abbreviations: IF = intermittent fasting; STF = short-term fasting; FMD = fasting-mimicking diet; CR = caloric restriction; CRP = C-reactive protein; IGF-1 = insulin-like growth factor-1; DSR = differential stress resistance; DSS = differential stress sensitization; QoL = quality of life; ↑ indicates an increase; ↓ indicates a decrease/reduction.

**Table 4:** Summary of key preclinical and clinical studies on ketogenic dietary strategies (KD) in oncology.

Author & Year [Ref]	Study Design	Tumor Type	Sample Size	Intervention Characteristics	Main Outcomes
**Talib et al., 2021** [58]	Preclinical molecular analysis	Multiple cancer types (review of mechanistic pathways)	n/a	KD and ketone bodies (BHB, acetone)	↓ IL-1β, ↓ TNF-α, ↓ IFN-γ; anti-inflammatory and potential anticancer actions.
**Shimazu et al., 2013** [57]	Preclinical (cellular/animal models)	Multiple tumor models	n/a	β-hydroxybutyrate (BHB) exposure	BHB suppressed oxidative stress via HDAC inhibition; anti-inflammatory and cytoprotective effects.
**Zhang et al., 2020** [59]	Preclinical (mouse model)	Colon cancer	n/a	KD intervention	↑ oxidative stress; ↓ MMP-9 expression; shift of TAMs from M2 → M1; antitumor activity.
**Said et al., 2014** [60]	Preclinical review	Colorectal cancer	n/a	KD-related metabolic modulation	Highlighted role of MMPs in tumor progression; KD may inhibit MMP-driven pathways.
**Elisia & Krystal, 2021** [61]	Preclinical/clinical integrative review	Various cancers	n/a	Low carbohydrate/KD strategies	Summarized anticancer mechanisms and limitations; highlighted preliminary evidence.
**Dmitrieva-Posocco et al., 2022** [62]	Preclinical (autochthonous CRC mouse model)	Colorectal cancer	n/a	KD-induced BHB; activation of HCAR2/HOPX	BHB inhibited tumor cell proliferation; suppressed CRC progression.
**Pflanz et al., 2019** [63]	Preclinical neuropharmacological study	Various tumor types	n/a	Ketone bodies modulation of NMDA signaling	NMDA pathway modulation; altered NAD^+^/NADH ratio; ↑ ROS → pro-apoptotic effects.
**Mundi et al., 2021** [64]	Translational review	Multiple tumors	n/a	KD metabolic mechanisms	Highlighted redox modulation and therapeutic potential in cancer.
**Fine et al., 2012** [65]	Pilot clinical trial	Advanced cancers (mixed)	10	4-week KD	Higher BHB associated with stable disease/partial remission; feasible & safe.
**Schmidt et al., 2011** [66]	Pilot clinical trial	Advanced-stage cancers	16	KD ± oil–protein supplementation	↓ weight; stable lipids; QoL improvements; no severe adverse events.
**Khodabakhshi et al., 2020** [67]	Randomized controlled trial	Breast cancer	60	MCT-based KD	↓ fasting glucose, ↓ weight, ↓ BMI, ↓ body fat; ↑ ketones; metabolic improvements.
**Plotti et al., 2020** [68]	Clinical study	Various cancers	n/a	Fasting + KD	↓ insulin; inverse correlation BHB–IGF-1; metabolic improvement.
**Lane et al., 2021** [69]	Clinical/translational review	Glioblastoma, breast, liver, lung, pancreatic, colorectal, head & neck	n/a	KD in adult cancer patients (≥18 years)	Improved survival, PFS, treatment response; QoL improvements in selected studies.

Notes: This table summarizes representative preclinical and clinical studies evaluating ketogenic dietary strategies (KD) in oncology. Sample sizes are reported only for human trials when available. Preclinical studies refer to murine or cell-based models. Outcomes reflect the principal antitumor, metabolic, inflammatory, or treatment-related effects reported by each study. Abbreviations: KD = ketogenic diet; LC = low carbohydrate; BHB = β-hydroxybutyrate; MCT = medium-chain triglyceride; MMP-9 = matrix metalloproteinase-9; TAMs = tumor-associated macrophages; HCAR2 = hydroxycarboxylic acid receptor 2; N = homeodomain-only protein homeobox; NAD^+^/NADH = nicotinamide adenine dinucleotide (oxidized/reduced); ROS = reactive oxygen species; QoL = quality of life; PFS = progression-free survival; ↑ indicates an increase; ↓ indicates a decrease/reduction.

**Table 5:** Representative studies on vitamin D, carotenoids, and cancer risk or prognosis.

Author & Year [Ref]	Study Design	Tumor Type	Sample Size	Main Findings
**Hossain et al., 2019** [76]	Systematic review and meta-analysis of observational studies	Breast cancer	Multiple cohorts (thousands of women)	Lower vitamin D status consistently associated with higher breast cancer risk.
**Bauer et al., 2013** [83]	Meta-analysis of prospective cohort studies	Breast cancer (postmenopausal)	5206 cases/6450 controls	Nonlinear inverse association between plasma 25(OH)D and breast cancer risk in postmenopausal women.
**Kim et al., 2021** [85]	Prospective cohort	Colorectal cancer (early-onset and precursors)	Large cohort of younger adults	Higher total vitamin D intake associated with reduced risk of early-onset CRC and precancerous lesions.
**Yuan et al., 2019** [90]	Prospective cohort (CALGB/SWOG 80405)	Advanced/metastatic colorectal cancer	1041 patients	Higher baseline 25(OH)D levels associated with better overall survival in metastatic CRC.
**Webb et al., 2015** [100]	Prospective cohort	Epithelial ovarian cancer	1631 women with EOC	Higher circulating 25(OH)D at diagnosis linked to improved survival in women with ovarian cancer.
**Leoncini et al., 2015** [107]	Systematic review and meta-analysis	Head and neck cancers (oral, pharyngeal, laryngeal)	16 epidemiological studies	Higher dietary carotenoid intake (β-carotene, β-cryptoxanthin, lycopene) associated with markedly reduced HNC risk.
**Hu et al., 2012** [114]	Meta-analysis of observational studies	Breast cancer	18 studies	Higher α-carotene intake significantly associated with reduced breast cancer risk; evidence for β-carotene less consistent.
**Han et al., 2022** [118]	Meta-analysis (22 studies)	Colorectal cancer	22 epidemiological studies	Higher serum/dietary β-cryptoxanthin, lycopene, α-carotene and β-carotene associated with lower CRC risk.
**Gallicchio et al., 2008** [119]	Systematic review and meta-analysis	Lung cancer	6 clinical trials + 25 observational studies	Overall modest inverse association between carotenoid intake and lung cancer risk; limited benefit from supplementation, especially in smokers.

Notes: Studies were selected for representativeness, methodological quality, and relevance to mechanisms discussed in the text. Abbreviations: 25(OH)D = 25-hydroxyvitamin D; CRC = colorectal cancer; EOC = epithelial ovarian cancer; HNC = head and neck cancer.

**Table 6:** Representative studies on physical activity, adiposity and cancer risk or prognosis.

Author & Year [Ref]	Study Design	Exposure/Intervention	Tumor Type/Outcome	Main Findings
**Friedenreich et al., 2010** [139]	Narrative review of epidemiological studies	Habitual physical activity	Multiple cancers	Summarized consistent inverse associations between higher physical activity and risks of several common cancers, supporting a dose–response relationship.
**Papadimitriou et al., 2020** [147]	Mendelian randomization analysis (UK Biobank)	Genetically proxied physical activity	Breast and colorectal cancer	Higher genetically predicted physical activity associated with reduced risks of breast and colorectal cancers, independent of BMI, supporting a causal role.
**Amirsasan et al., 2022** [157]	Systematic review and mechanistic analysis	Physical activity (various domains)	Colorectal cancer	Numerous clinical trials and meta-analyses have demonstrated that exercise significantly reduces the risk of colorectal cancer, supporting its role as an effective preventive strategy.
**Moore et al., 2016** [161]	Prospective pooled cohort (~1.44 million adults)	Leisure-time moderate-to-vigorous physical activity	26 cancer sites	Higher leisure-time activity associated with lower risks of 13 cancers (including breast, colon, endometrial, kidney and bladder), with risk reductions up to ~20–25%.
**Schmid et al., 2015** [159]	Systematic review and meta-analysis (33 studies)	Total and recreational physical activity	Endometrial cancer	Regular physical activity associated with ~20% lower risk of endometrial cancer; findings robust across study types and adjustment models.
**Psaltopoulou et al., 2016** [158]	Systematic review and meta-analysis (10 cohort, 12 case–control studies)	Leisure-time and occupational activity	Gastric cancer	Higher levels of physical activity associated with ~19% lower gastric cancer risk compared with lowest activity categories.
**Behrens et al., 2014** [160]	Systematic review and meta-analysis	Physical activity (various domains)	Gastroesophageal cancers	Greater physical activity associated with lower risk of esophageal and gastric cardia cancers, supporting a protective role beyond weight control.
**Cataldi et al., 2021** [128]	Systematic review of clinical trials	Structured exercise training during/after therapy	Cancer-related fatigue	Exercise interventions consistently reduced cancer-related fatigue and improved functional capacity across heterogeneous cancer populations.
**Fischetti et al., 2019** [131]	Non-randomized exercise intervention	Supervised multicomponent training	Lymphoma patients	Program improved physical fitness and psychological well-being in lymphoma patients undergoing or post-treatment, supporting exercise oncology in hematologic cancers.
**Patel et al., 2019** [164]	ACSM roundtable (expert consensus)	Physical activity and sedentary behavior recommendations	Cancer prevention and survivorship	Concluded that regular aerobic and resistance exercise reduces cancer risk and improves treatment tolerance and survivorship outcomes; provided clinical prescription guidance.
**Figlioli et al., 2025** [136]	Systematic analysis (Global Burden of Disease Study 2021)	High body-mass index (BMI)	Multiple cancers	Estimated that elevated BMI accounted for ~356,700 cancer deaths and 8.89 million DALYs globally in 2021, with increasing trends since 1990 and strongest associations for colorectal and pancreatic cancers.
**Hidayat et al., 2018** [170]	Systematic review and meta-analysis of observational studies	Early-life body fatness	8 adult cancer types	Higher body fatness at young ages associated with increased risk of several adult cancers, indicating long-term carcinogenic effects of childhood and adolescent adiposity.
**World Cancer Research Fund/AICR, 2018** [137]**; 2023 update** [171]	Continuous Update Project reports	Body fatness, weight gain, physical activity, sedentary behavior	Multiple cancers	Concluded that excess body fat and weight gain convincingly increase risk of several cancers, while regular physical activity convincingly or probably decreases risk for breast, colorectal and other sites.

Notes: Studies were selected to illustrate both preventive and survivorship-related effects of physical activity and the impact of adiposity/body fatness on cancer risk and prognosis. Abbreviations: BMI = body mass index; WHO = World Health Organization; WCRF/AICR = World Cancer Research Fund/American Institute for Cancer Research.

**Table 7:** Representative evidence on complementary and mind–body approaches in oncology.

Author & Year [Ref]	Study Design/ Sample	Cancer Type	Intervention	Main Findings
**Carlson et al., 2017** [9]	Overview of RCTs and observational studies	Breast, prostate, hematologic	Mind–body therapies (meditation, yoga, Tai Chi)	Improvements in fatigue, anxiety, depression, and QoL; moderate evidence for symptom management.
**Cramer et al., 2022** [10]	Systematic review of mind–body interventions	Multiple cancers	Yoga, Tai Chi, Qigong	Consistent reduction in cancer-related fatigue and stress; mixed evidence for pain and sleep.
**Deleemans et al., 2023** [11]	Review of recent clinical trials	Breast, gynecologic	Mind–body and integrative practices	Benefits for fatigue, sleep, QoL; growing evidence for immune-modulating effects.
**Horneber et al., 2012** [12]	Systematic review & meta-analysis; >30 studies	All cancers	General CAM use (herbal medicine, supplements)	33–47% of patients use CAM; variable quality; potential for harmful herb–drug interactions.
**Frenkel et al., 2018** [13]	Narrative review	Lung cancer	Integrative medicine & supplements	Some supplements pose risk of interaction; CAM helpful for symptom relief with supervision.
**David et al., 2021** [14]	Review on CRF	Multiple cancers	Integrative & mind–body therapies	Mind–body therapies effective for reducing CRF; limited but promising evidence.

Notes: The table includes key studies evaluating symptom management, psychological outcomes, and treatment-related effects across different tumor types. Abbreviations: QoL = Quality of Life; CRF = Cancer-Related Fatigue; CAM = Complementary and Alternative Medicine; RCT = Randomized Controlled Trial.

## Data Availability

No new data were created or analyzed in this study.

## Supplementary Materials

The following supporting information can be downloaded at: https://www.mdpi.com/article/10.3390/pathophysiology32040070/s1, Table S1: Summary of the Literature Search Approach.
