# Supplementary material for: Lifestyle-Based Approaches to Cancer Prevention and Treatment: Diet, Physical Activity, and Integrative Strategies
Source: Pathophysiology. 2025 Dec 17;32(4):70. doi: 10.3390/pathophysiology32040070 (PMC12735890; doi:10.3390/pathophysiology32040070)
Supplement: Supplementary file 1 [file pathophysiology-32-00070-s001.zip › pathophysiology-3953279-supplementary.pdf]

Supplementary Table S1
Summary of the Literature Search Approach

Database	Search terms (main concepts)	Time frame	Search completion date	Records screened	Records considered relevant
**PubMed**	cancer; oncology; lifestyle; diet; nutrition; physical activity; exercise; obesity; overweight; complementary therapies; integrative oncology; carcinogens; tobacco; smoking; alcohol; radiation; fasting; caloric restriction; ketogenic diet; vitamins; carotenoids; major cancer types	2001–2025	Search completed on 11 Aug 2025	820	214
**Scopus**	cancer AND lifestyle; diet; exercise; obesity; CAM; carcinogens; fasting; ketogenic diet; vitamins; carotenoids; specific cancer types	2001–2025	Search completed on 11 Aug 2025	640	176
**Google Scholar** (manual search)	lifestyle and cancer; diet and cancer risk; exercise oncology; integrative oncology; nutrition and malignancies	2001–2025	Search completed on 11 Aug 2025	~500	92

Note: The search was conducted iteratively across the included databases, with final consolidation and completion on 11 August 2025.

This table provides a general overview of the search approach adopted for the narrative review. It is not intended to represent a systematic or PRISMA-based selection process.
